# The gap that Matilda will bridge: a look at the Colombian case

**DOI:** 10.12688/f1000research.147674.1

**Published:** 2024-06-06

**Authors:** Isabel Cristina Rivera-Lozada, Andrés Mauricio Gómez-Sánchez, Oriana Rivera-Lozada

**Affiliations:** 1 Departamento de Economía, Universidad del Cauca, Popayán, Cauca, Colombia; 2 Facultad de educación, Universidad Nacional Mayor de San Marcos, Lima, Peru

**Keywords:** Gender gap, segregation, discrimination, scientific research, STEM.

## Abstract

**Objective:**

To determine gender gaps in Emeritus researchers in Colombia.

**Methods:**

Oaxaca-Blinder-Kitakagwa decomposition model, correcting the sample selection bias with the inclusion of Mills’ inverse ratio (Heckman’s Lambda) through an ordered *probit* model. Data: Information available in the ScienTI Platform – Colombia during the period 2015-2021.

**Results:**

The results show that the gender gap between female and male researchers is 5.8%. To achieve Emeritus status, one must be over 65 years old, and the possibility of achieving Emeritus status is 5.1% higher for female researchers than for their male counterparts. These differences can be explained by the time constraints that female researchers face in being productive, as they spend more time than male researchers on caregiving responsibilities, either due to motherhood or the care of other dependent family members.

**Conclusions:**

The results obtained allow us to affirm that there is a gender gap in scientific research in Colombia in the Emeritus research category in the calls for proposals for the period 2015-2021. Moreover, the existing gap cannot be explained by factors associated with attributes of education and academic productivity that are part of the regulatory requirements, insofar as not being explained by them, it evidences the existence of discrimination against women researchers to access the highest research category.

## Introduction

Gender equality, beyond being a fundamental human right proclaimed by the United Nations in the Sustainable Development Goals, is a decisive factor for justice and social progress ( Naciones Unidas, s/f, 2020). In the context of the fourth industrial revolution, the participation of women in science, research and technology is one of the lowest in the world and in Latin America. In Colombia, 33.2% of the total number of people employed in these sectors are women ( Chiarella et al *.*, 2023).

Gender gaps in science, research and technology respond to multiple factors that include low participation in the sector, caused by segregation processes resulting from the traditional assignment of roles that lead to broken ladders and sticky floors ( Rivera-Lozada et al., 2023a), as well as phenomena such as the Matilda effect where sexism in science has been a source of invisibilisation and exclusion of women ( Saborit-Rodríguez et al., 2022) or the Curie effect that has intimidated female scientists in the face of such a high figure to emulate ( Des Jardins, 2010).

Recently, several research have sought to identify the existence of gender differences in research authorship ( Pinho-Gomes et al., 2020) or research productivity linked to gender ( Mayer & Rathmann, 2018). In general, it is possible to document the gender disparity in science in terms of productivity and the recognition derived from it ( Sá et al., 2020).

In the Colombian case, studies related to gender gaps in research ( Ávila-Toscano et al., 2019; Gutiérrez, 2012; Tovar Rojas, 2008) have not explored yet the existence of gaps in access to research rankings granted by the Ministry of Science, Technology and Innovation (MinCiencias): Junior, Associate, Senior and Emeritus. The requirements for classification in the aforementioned categories respond to strictly academic criteria previously stipulated ( MinCiencias, 2021) and therefore the consideration of discriminatory elements of gender or any other kind is not pertinent. As a result, female researchers have the same possibility of accessing the Emeritus category as male researchers. Therefore, if the above is not met, we are faced with phenomena such as the Matilda effect or the Curie effect as a possible explanation for the results.

In this perspective, this research has the objective to identify the existence of gender gaps in the Emeritus category as it is the most demanding, with the highest research level and with a small number of male and female researchers nationwide. This allows filling an analytical gap by determining the possible existence of gender gaps to achieve the Emeritus research category, taking into account the profile of the group of researchers of MinCiencias for the 2015-2021 period attributable to gender discrimination identified in the scientific field.

This research brings for the first time the analysis of gender discrimination in the highest research category, at least for Colombia, and the inclusion of Heckman’s Lambda to deal with the sample selection problem through an ordered Probit model and not with a Probit model, as is the tradition to capture the different hierarchies in the categories of researchers. The overall results reveal a gap of 5.1%, whose difference in the coefficients attributable to gender discrimination phenomena is increasing from 4% in 2015 to 4.6% in 2017, remains the same for 2019 and reaches 4.8% in 2021.

## Literature review

The gender gap is the form used to represent the disparity between men and women in terms of resources, opportunities, access and rights ( CEPAL, s/f; Sebastiá, 2020). In the case of Science, Technology and Innovation (STI), these gaps are difficult to measure because there is little available internationally data and indicators to study these phenomena ( López-Bassols et al., 2018) that some find associated with career choice by men and women. In the case of women, it may be related to stereotyped occupations ( Morales Inga & Morales Tristán, 2020) as a consequence of what appears to be a natural extension of the traditional roles of patriarchal societies where women choose occupations related to care, such as nursing, education, childcare, while men choose professions related to authority such as military, judicial, legislative and engineering careers, among others ( Rivera-Lozada et al., 2023a).

To understand the issue of the status of women in science, technology, engineering and mathematics (STEM), research in the United States identified factors such as motivation, self-concept, self-efficacy and identity, biases, stereotypes, university culture and lived experiences, barriers to tenure, promotion, work-life balance and administrative advancement ( Blackburn y Heppler, 2020) in an effort to capture environmental and personal factors attributable to the profile.

The literature review showed that the determining factors of scientific production include geographical location ( Luna Morales y Luna Morales, 2018; Pinho-Gomes et al., 2020), position ( Castro, 2018; Pons Peregort et al., 2013), time commitment ( Maceira Ochoa, 2017; Pinho-Gomes et al., 2020) research classification or ranking ( Mayer y Rathmann, 2018), marital status ( Aiston y Jung, 2016; Sougou et al., 2022), number and age of children ( Pons Peregort et al., 2013), inclusive funding programmes ( López Belloso y Díez Sanz, 2017; Montané y Carvalho, 2012) institutional determinant ( Gomez, 2019; Moreno Cubillos et al., 2012; Ortega Ayala, 2019), sexist bias ( Guil Bozal, 2016; Sougou et al., 2022), gender stereotypes and roles ( Bordons et al., 2013; García-Jiménez y Herrero, 2022), discrimination ( Truffa, 2012), income level ( Churba, 2023; Jabbaz et al., 2019), segregation ( López Montes y Morón Gallego, 2017; Vargas et al., 2016), glass ceiling ( Cárdenas Tapia, 2015; García, 2014; Rivera Lozada et al., 2020) and recognition ( Matilla Quiza y Mo Romero, 2014; Sá et al., 2020).

From this review emerges the classification of research barriers that contribute to gender gaps in four categories: i) Academic offer in terms of fields of knowledge, access, participation and presence of women, ii) Research policy as a guarantor or not of inclusion through funding strategies, participation and promotion of relevant research lines with a female perspective, iii) Scientific production as a result of the increase in the academic offer, the increase in female enrolment in higher education, specifically in STEM, the strengthening of research groups and the increase in scientific collaborations, and finally, iv) Profile based on characteristics, motivations and distinctive features of female and male researchers ( Rivera-Lozada, et al., 2023b; UNESCO, 2019).

This research addresses the factors associated with the fourth category, Profile, abandoning the diversity of factors proposed in the other categories, not because they are considered less important, but because of the difficulty in measuring them and the availability of the corresponding information. Thus, the study incorporates age, classification of the researcher, place of birth, affiliation, level of education and gender, in order to show, based on them, the existing gaps in the probability of a male or female researcher reaching the highest research category in Colombia.

## Methods

### Design and population of the study

The research has a quantitative approach developed through stochastics models. Specifically, Oaxaca-Blinder-Kitakagwa decomposition modelling is implemented, correcting the sample selection bias with the inclusion of Mills’ inverse ratio (Hackman’s Lambda) through an ordered *probit* model. The population of the study includes all university professors and/or researchers registered in the Ministry of Science, Technology and Innovation in Colombia.

### Sampling type and size

The data used in this study are drawn from MinCiencias database spanning in four waves (2015, 2017, 2019, and 2021) with 77,168 observations. It is worth mentioning that waves in previous periods are not taken into account, given that the Emeritus category had not been achieved by any researcher presented to that date, probably due to the requirement that this implied. The data is openly accessible in the ScienTI Platform. ^1^

### Procedures

The stochastic model used explains the differences in the probability of achieving the Emeritus research category between men and women, and specifically how much of this gap comes from observable and unobservable variables attributable to gender discrimination.

For this purpose, an Oaxaca-Blinder-Kitakawa decomposition model ( Blinder-Kitakawa, 1973; Oaxaca, 1973) is implemented within the variant of Fairlie (1999), 2005 and Yun (2004), including for the first time in gender studies, dichotomous variables to capture gaps in different research categories, using therefore a discrete choice Probit model. It is worth mentioning that this model also introduces Heckman’s Lambda (1979), also known as Mills’ inverse ratio, to deal with possible of sample selection bias problems, since possibly not all researchers in Colombia are registered with MinCiencias and therefore the sample would not be random. The novelty with this sample selection correction is that the model used is an ordered Probit, to capture the hierarchy of the different research categories.

Since this study uses data from 2015 onwards, it is not possible to implement panel data models as there are so few years (T=4 periods) and an increasing number of individual units (N); then (N>T): and according to Pesaran (2015), the estimators of fixed and random effects, instrumental variables and the generalised method of moments (GMM), will be biased. For this reason, this study has conducted a cross-sectional analysis for each period and compares the results over time. ^2^

In formal terms, the model assumes that the dependent variable (the differentials in the probability of obtaining the Emeritus status between men and women) is a dichotomous variable explained non-linearly for a set of covariates. In this sense, following Fairlie, (1999) and Vicéns-Otero (2012), the model in general is as follows:

$${\bar{Y}}_{H}-{\bar{Y}}_{M}=\left[\sum_{i=1}^{N_{H}}\frac{F\left(X_{\mathrm{Hi}}{\hat{\beta }}_{H}\right)}{N_{H}}-\sum_{i=1}^{N_{M}}\frac{F\left(X_{\mathrm{Mi}}{\hat{\beta }}_{M}\right)}{N_{M}}\right]+\left[\sum_{i=1}^{N_{M}}\frac{F\left(X_{\mathrm{Mi}}{\hat{\beta }}_{H}\right)}{N_{M}}-\sum_{i=1}^{N_{M}}\frac{F\left(X_{\mathrm{Mi}}{\hat{\beta }}_{M}\right)}{N_{M}}\right]$$ (1)

Where ${\bar{Y}}_{H}$ and ${\bar{Y}}_{M}\;$ are the average probabilities of achieving the emeritus status for men and women respectively. On the other hand, F(∙) denotes the cumulative distribution function of the normal distribution evaluated at $X\hat{\beta }$ for both groups. The first bracket on the right side captures the observable factors that explain the probability differentials in both groups. The second bracket also explains the differentials, but due to unobservable factors, attributable to discrimination phenomena. Lastly, $N_{M}$ and $N_{H}$ are the number of observations for men and women, respectively.

As an example, the existence of a 10% probability differential between men and women to achieve the highest category, and in addition, 7% of this percentage is attributable to observable characteristics (such as level of education or university of origin, among others), the remaining 3% is attributed to gender discrimination because it is not explained by any other observable factor.

For this study, the stochastic model is based on human capital models ( Schultz, 1959) and on Mincer’s (1974) extended income model, where education and experience/age are key determinants of the phenomenon to be explained, but also another set of covariates associated with the individual. In this sense, it is assumed that the probability differentials in achieving the maximum category are attained according to a set of observable characteristics, where most of them come from the researcher’s background and a few others are of an institutional and statistical nature (Heckman’s Lambda).

Specifically, the explanatory variables X ( equation 1), the age of the person is considered as a proxy variable for work experience. This happens because the database used does not have this information, but fundamentally because, as stated by ( Zveglich et al., 2019), when calculating experience in the traditional way by subtracting the years of education minus five from age (assuming that most children start primary education at that age), this value is the same for all individuals of the same age and educational level, regardless of gender. Therefore, age is included in this study with the understanding that the older the age, the more experience, but also because the Emeritus category is only available for researchers who are 65 years of age or older according to current regulations. It is worth noting that age squared is also considered to capture the concavity of the function in this variable, that is, to check if the probability differentials of being an Emeritus researcher between the two groups grow at decreasing rates with age ( Gómez-Sanchez y Ramírez-Gutiérrez, 2021).

On the other hand, the Mincer equations include the level of academic education because the higher the level of study, the higher the salary earned. However, they are included here due to current regulations, since the highest categories require more advanced qualification levels; thus, the Emeritus category requires, but not limited to, a doctoral level of academic education. At this point it is worth noting that the levels of postdoctoral education are not academically recognised by MinCiencias, as they are international professorial stays whose objective is to carry out academic research. Therefore, according to Resolution 1764-2013 of MinCiencias they are not study programmes and do not lead to a degree. In this sense, only doctoral studies will be taken into account.

The stochastic model assumes that other characteristics also explain the possibility of obtaining the highest category, such as the area of academic education, the university of origin and nationality. These variables are, in this study, part of the extension of Mincer’s (1974) traditional model.

The area of training is important because depending on the availability of financial resources, evolution of technology, innovations and culture, some fields of science have a greater amount of research than others ( Ríos Gómez & Herrero Solana, 2005), as is the case in the last decade of research on artificial intelligence, biotechnology, renewable energies and neuroscience leading research worldwide ( Banich & Compton, 2018; Clark & Pazdernik, 2015; Russell et al., 2015). Therefore, belonging or not to these areas has an impact on the possibility, quantity, and quality of research and thus on obtaining the highest category. For this reason, the stochastic model controls this situation by introducing the researcher’s area of knowledge.

The affiliation of researchers is also relevant, and, in the case of Colombia, it is concentrated in the universities in the centre rather than in the periphery of the country. This is due, as mentioned above, to the academic quality of the universities and the financial resources available or accessible to them, or, as stated by Ríos Gómez and Herrero Solana (2005), to the industrial, economic and political development of some regions compared to others. In this sense, the most important universities in the country are located in the golden triangle of Colombia, which corresponds to the cities of Bogota, Medellin and Cali, where the most important universities in the country are located (Universidad Nacional, Javeriana, Andes, Antioquia and Valle).

On the other hand, the nationality of the researcher could also explain the phenomenon analysed, since the possibility of having a doctorate, speaking a foreign language, or having authored articles published in international journals, among others, is high. Some of these researchers have been hired directly, others have arrived under the figure of visiting professors in the context of international academic mobility at universities, but they have stayed in the country and have become part of MinCiencias’ calls for proposals.

Furthermore, to account for the possibility that the area of education is related to the university of origin, a dichotomous interaction variable is also included. Finally, as the sample may not be random, the estimators will be biased and inconsistent if estimated using the Ordinary Least Squares (OLS) method. Therefore, Heckman’s Lambda is included within the explanatory variables X in equation 1, as a way to avoid endogeneity due to omitted variable. However, this process involves implementing a stochastic model prior to equation (1) to obtain the variable in question.

Heckman’s Lambda is traditionally obtained through a Probit model to capture the participation of the individual, in this case, of the Colciencias ranking. Nevertheless, because in this case the variable analysed has four categories with a hierarchical order (Emeritus, Senior, Associate and Junior), then the dependent variable is ordinal, and therefore an ordered Probit model must be used. In this sense, the model allows finding the probability of observing the result i corresponding to the probability that the estimated linear function plus the random error is within the critical points that separate the research categories:

$$\mathrm{Pr}\left(z_{j}=i\right)=\mathrm{Pr}\left(k_{i-1}<\sum_{i=1}^{k}\alpha_{k}x_{\mathrm{kj}}+u_{j}\leq k_{i}\right)$$ (2)

Where k are the critical points; i refers to the four research categories, $\alpha_{k}$ are the parameters to be estimated; $x_{\mathrm{kj}}$ are the variables that explain participation. This includes age, an interaction variable between age and university of origin, and another interaction variable between area of training and university of origin. The variable $u_{j}$ are the stochastic errors which follow a normal distribution.

Once the model is estimated ( equation 2), the default value of ${\hat{z}}_{j}$ , is obtained, which is replaced in the normal density function $\phi \left({\hat{z}}_{j}\right)$ and in the cumulative distribution function $\Phi \left({\hat{z}}_{j}\right)$ ; and finally, its division is the Heckman’s Lambda or Mills’ inverse ratio:

$${\hat{\lambda }}_{H}=\frac{\phi \left({\hat{z}}_{j}\right)}{\Phi \left({\hat{z}}_{j}\right)}=\frac{\frac{1}{\sqrt{2\pi }}e^{-\left({{\hat{z}}_{i}}^{2}/2\right)}}{\int_{-\infty }^{{\hat{z}}_{i}}\frac{1}{\sqrt{2\pi }}e^{-\left({{\hat{z}}_{i}}^{2}/2\right)}}$$ (3)

With all the above, and taking into account equations (1) and (3); the stochastic model specifically to determine the gender gaps in the Emeritus category has the following structure:

$$Y=\left\{ \begin{array}{cc} 1 & [\beta_{1}{\text{edad}}_{i}+\beta_{2}{\text{edad}}_{i}^{2}+\beta_{3}{\text{nivel}}_{i}+\beta_{4}{\text{area}}_{i}+\beta_{5}{\mathrm{nac}}_{i}+\beta_{7}{\text{univ}}_{i}+\beta_{6}d2_{i}+\beta_{8}{{\hat{\lambda }}_{H}}_{i}+\varepsilon_{i}]\geq 0\\ 0 & o.c \end{array}\right.$$ (4)

The variable *Y* is dichotomous and takes the value of 1 if the researcher is in the Emeritus category and 0 otherwise. The explanatory variables are age ( *age*), age squared ( *age* ^2^); academic level ( *level*), area of knowledge ( *area*), nationality ( *nat*), and university of origin ( *univ*). Moreover, d2 is the dichotomous variable that captures the interaction between the area of education and university of origin; and ${\hat{\lambda }}_{H}$ is the Heckman’s Lambda estimated in equation 3. Finally, $\varepsilon_{i}$ are the random errors that are assumed to follow a normal distribution and are well-behaved; for example, $\varepsilon_{i}∼N\left(0,1\right).$ ^3^

### Variables

**Figure 1.**
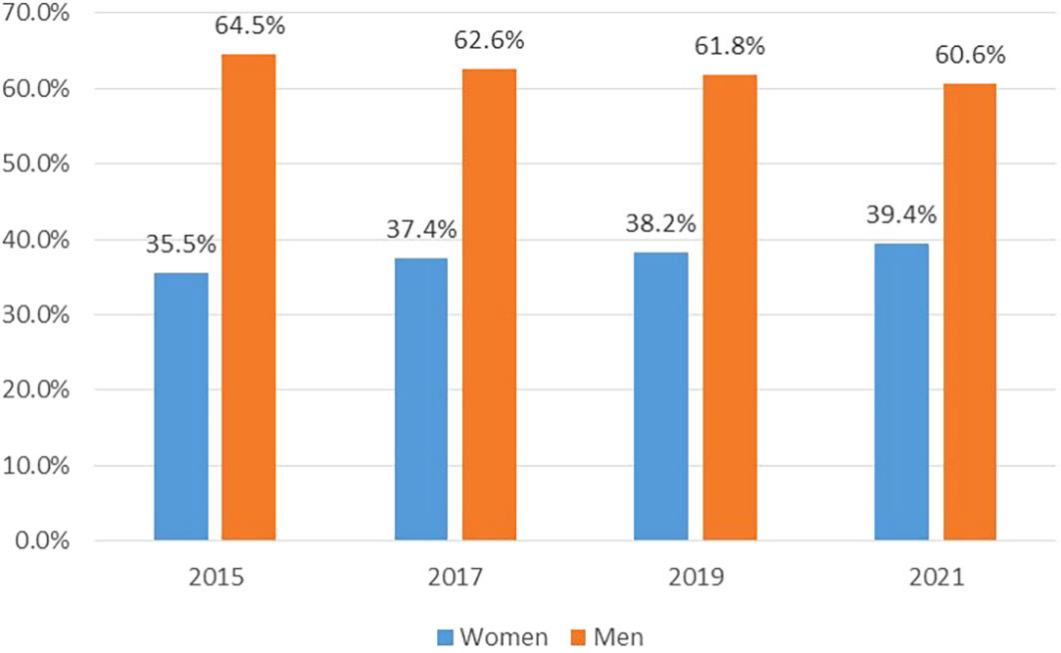
Proportion of men and women by call for applications. Source: Own elaboration.

Since this study attempts to show gender gaps between men and women in terms of research categories, Figure 1 shows the proportion of people who applied to MinCiencias’ call for proposals in the analysed period, broken down by men and women.

The figures reveal that the number of women who have applied to the calls for proposals has always been lower compared to the group of men. Even though the participation of women is increasing, from 35.5% in 2015 to 39.4% in 2021, this change has been very subtle. On the other hand, men participation has been decreasing, but also only slightly, from 64.5% in 2015 to 60.6% in 2021. This allows us to affirm two things. First, there are still gaps that are stable over time, at least for researchers who apply to these calls. Second, while these figures do not reveal any kind of ex ante discrimination, they could indicate in some way that the number of female researchers in Colombia is lower than that of men, although it must be borne in mind that possibly not all researchers are included in the calls for proposals.

As the objective of the study links gender with the highest research category, Table 1 shows the evolution of the Emeritus category, classified by gender over the years in which the calls were made.

**Table 1.** Share of other aspects related to research.

	2015		2017		2019		2021	
	Men	Women	Men	Women	Men	Women	Men	Women
PhD.	2.62%	2.19%	50.10%	49.09%	49.73%	48.28%	47.28%	45.20%
Social Sciences	23.92%	30.93%	26.49%	34.10%	27.99%	35.72%	26.58%	33.68%
Age	44.89	43.88	44.95	43.82	44.75	43.82	44.87	43.89
University	11.71%	14.15%	10.81%	12.60%	10.31%	11.25%	9.04%	9.50%
Nationality	92.87%	94.53%	93.70%	95.02%	94.11%	95.51%	94.87%	96.09%
Observations	6,479	3,563	8,132	4,856	10,374	6,409	12,773	8,296

Source: Own elaboration.

**Figure 2.**
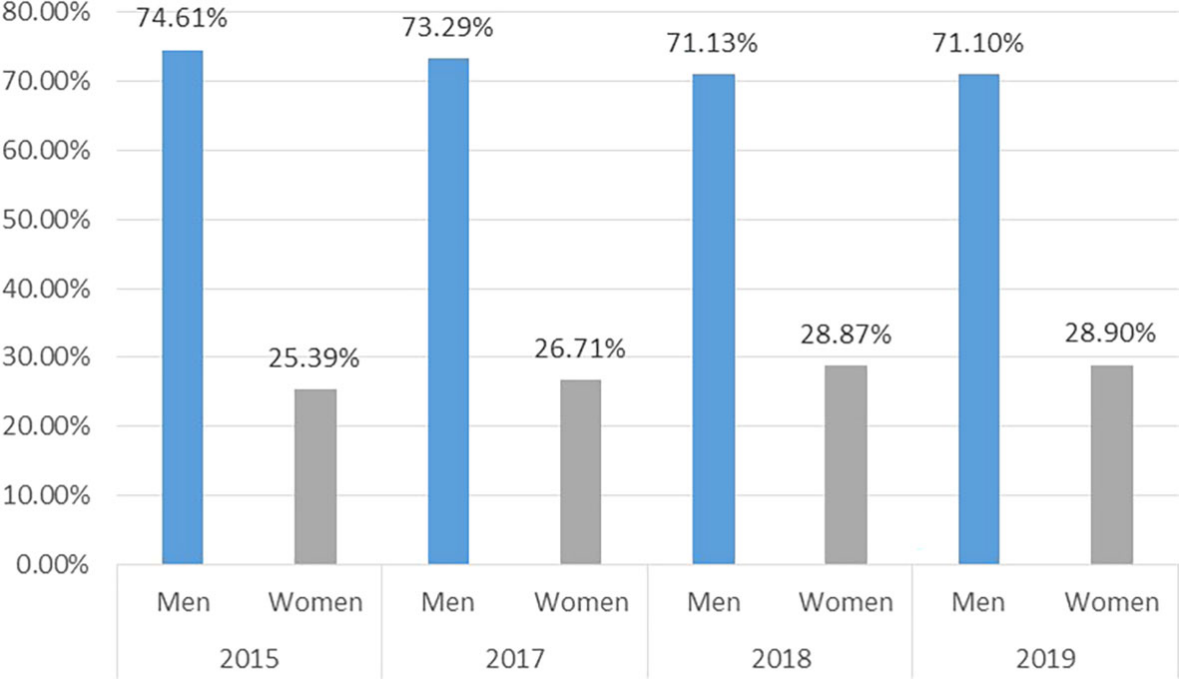
Gender proportion of researchers in the Emeritus category. Source: Own elaboration.

The figures show that in the Emeritus category ( Figure 2), it is historically men and not women who can achieve this recognition. In fact, in 2015 the number of women who obtained this category out of the total represented 25.39% and by 2021 it was only 28.90%, that is, an absolute increase of 3.3%, while in the case of men, the proportion has decreased almost imperceptibly, from 74.6%1 in 2015 to 71.1% in 2021. This indicates that the gap remains in Colombia, and for every three men in this category there is only one woman.

**Figure 3.**
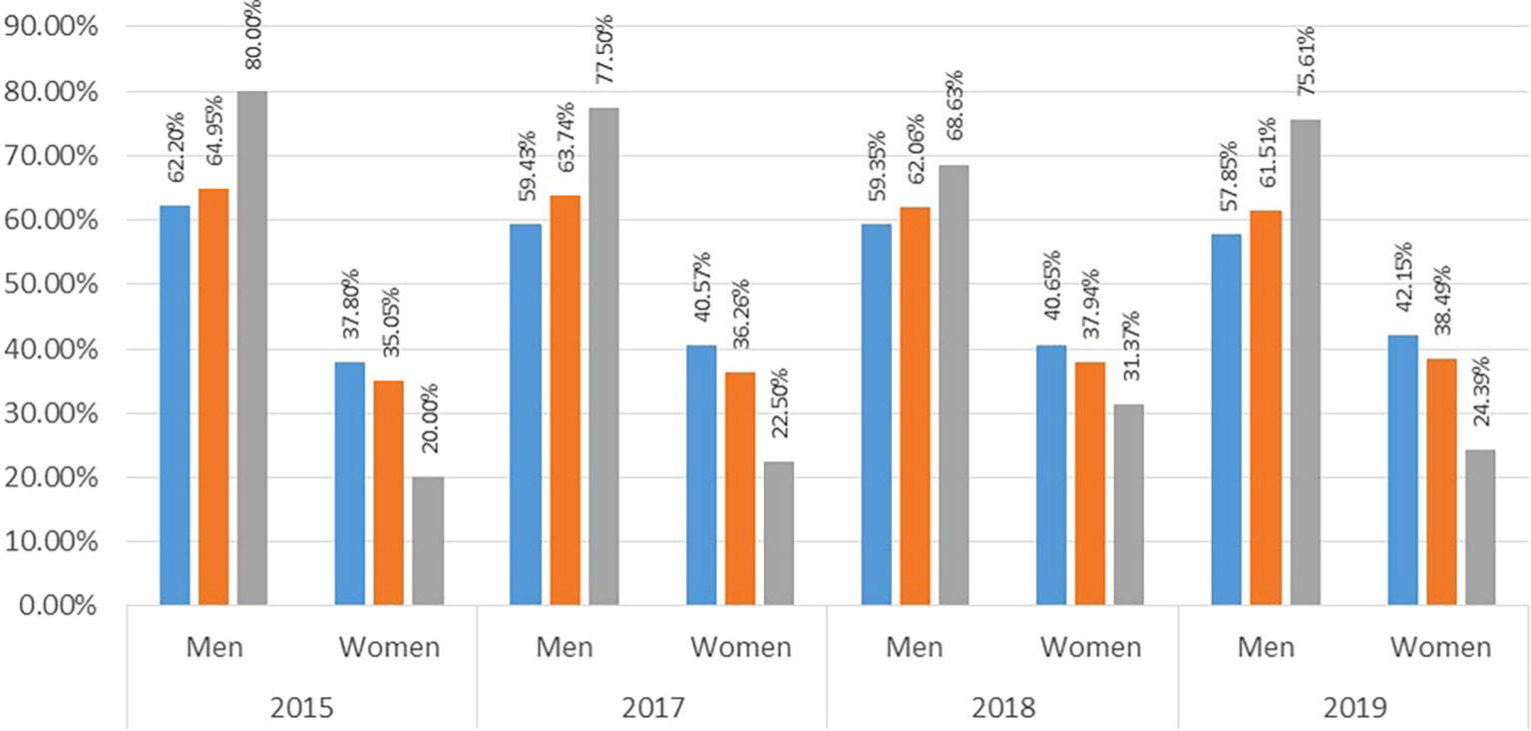
Proportion of male and female researchers in Senior, Associate and Junior categories. Source: Own elaboration.

It is worth noting that in the remaining categories below the maximum (Senior, Associate and Junior), the pattern is also repeated. As Figure 3 shows, in the Senior category, the proportion of men exceeds that of women, regardless of the period or call for applications, at around 60%, while for women it is only 40% on average. In the case of the Associate category, the situation is similar, with men accounting for 63% and women for 37% on average. Finally, in the Junior category, the most recurrent of all, but at the same time the lowest or least demanding, 75% of the people in this category are men and the remaining 25% are women.

With all of the above, it can be inferred that female participation in the different research categories in Colombia is less than half compared to that of men. In this order of ideas, it does not seem to be a simple coincidence that there are such high differences across the categories, because there are regulations defined by MinCiencias to obtain them. In this sense, it is necessary to know what determines these gaps, whether they are observable factors (such as academic education levels or university of origin, for example), or whether there are unobservable factors associated with discriminatory phenomena, especially in the highest category (Emeritus), given that it is the most demanding to achieve among all.

Other relevant aspects that could help to investigate this phenomenon are listed in Table 1. Given that the highest research categories require higher levels of academic education (specifically doctoral education), the figures show that the participation of researchers with PhD degrees in the different calls for applications has been similar between men and women, although it has always been slightly higher for men. It is also worth noting that this participation has been decreasing for both genders. Indeed, between 2017 and 2021 it has decreased by approximately 4% and 3% for women and men, respectively.

From the classification of sciences: natural, engineering and technology, medical and health, agricultural, social and humanities (OECD, 2007), the majority of researchers in Colombia (around 30%) come from the social sciences. In this area, female participation has been slowly increasing from 30.93% in 2015 to 33.68% in 2021. It should be noted that participation has always been higher for women than for men, where the average differential has remained below 7% in the period analysed.

**Figure 4.**
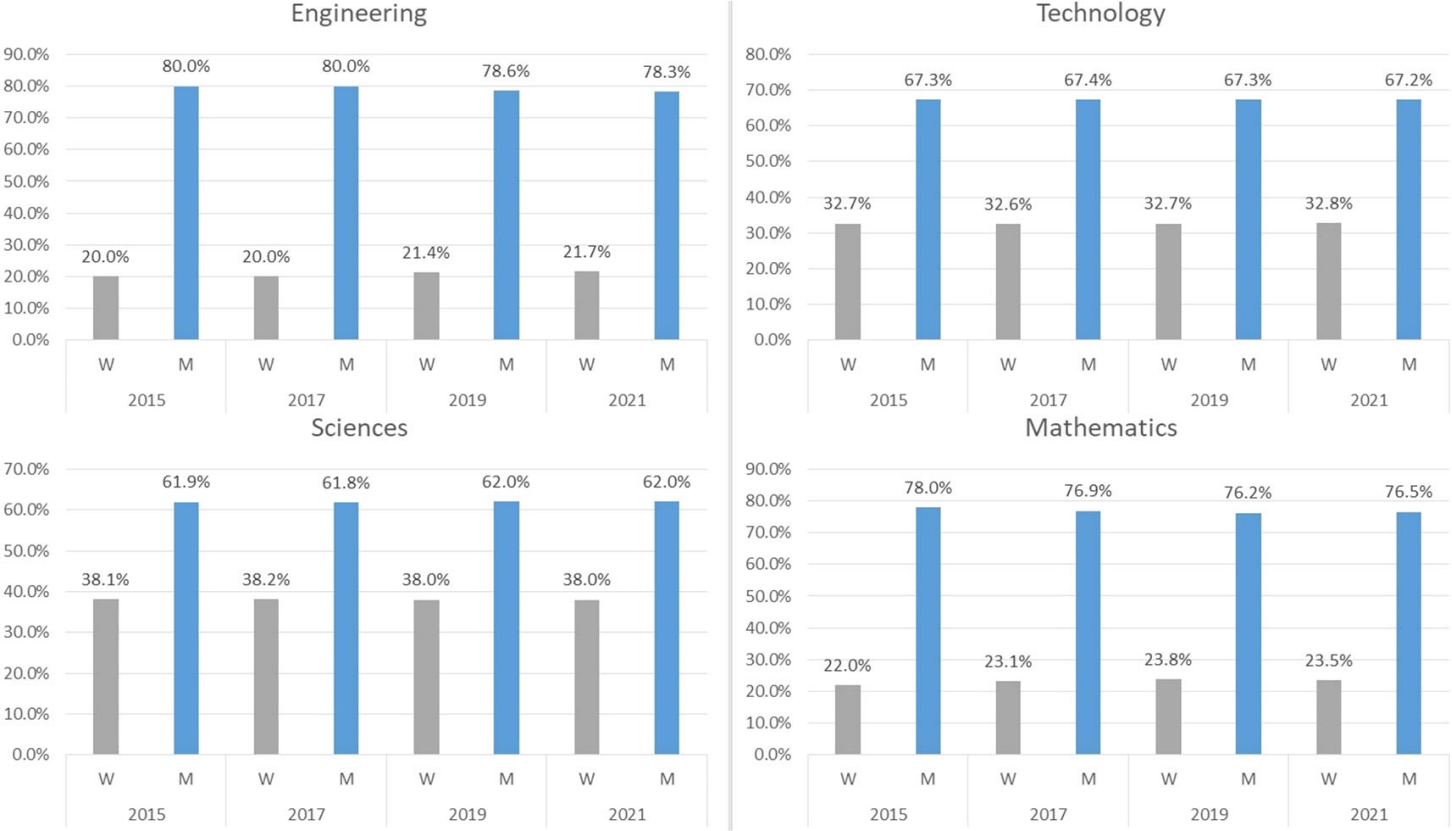
Gender gaps according to STEM categorisation. Source: Own elaboration.

The knowledge society also marks a direction in which new gender gaps are opening up, and this is what is happening with research in STEM (science, technology, engineering and mathematics) areas, Figure 4 shows the recent evolution of gender gaps in this area for Colombia.

The figures reveal that, in research areas such as mathematics and engineering, the gap between men and women is too wide (close to 60% on average in both cases) and remains almost constant over the period under analysis. In the case of science and technology, the gap is less marked, close to 25% on average for both cases; however, it also tends to remain unchanged over time.

The above indicates that in all STEM research areas for Colombia in its recent academic history there is male hegemony. However, there are some fields of knowledge where this phenomenon is more recalcitrant, such as mathematics or engineering, where approximately for every four men there is only one woman doing research, and others where this ratio is 2:1.

To achieve Emeritus status, the requirements include, but are not limited to, being over 65 years of age. The information in Table 1 shows that the average age between men and women is very similar (men around 44 and women around 43). The histograms of age by gender and year (not shown) reveal a distribution similar to a Chi-square for each call. In this sense, there are few cases of people under 40 years of age; however, from this age up to 60 years of age there is a large number of researchers; finally, after this age range, the proportion is decreasing.

On the other hand, if the university of origin is Universidad Nacional de Colombia, Los Andes, Javeriana, Antioquia or Valle, the figures show that participation is higher for women than for men at the beginning of the sample period (in 2025 it was 11.71% for men and 14.15% for women) but in the last call for applications in 2021 the gap is much smaller (9.04% versus 9.50%, respectively). It is also evident that the origin of these universities has been decreasing regardless of gender, which reveals that other universities have begun to gain prominence in research in Colombia, as is the case of some universities on the Colombian Atlantic coast, such as the Universidad de la Costa, Universidad Del Norte and Universidad de Cartagena.

In terms of nationality, as might be expected, the majority of the participants in the call for applications are of Colombian origin (around 95%). It is also worth noting that there is a very subtle differential in foreign nationality between men and women, as overall 6% of men are non-Colombian, while for women it is 5%. Unfortunately, the database does not provide exact information on the country of origin, only whether they are Colombian or foreign.

### Statistical analysis

STATA Version 17.0 (StataCorp. 2021, StataCorp LLC.) (RRID: SCR_012763) software is used for the data statistical analysis.

Table 2 shows the estimates of the stochastic model ( equation 4). The first column corresponds to the results for the 2015 call, the second for the 2017 call and so on. The last column shows the results for all calls together. In general, the results show that the parameters are positive and statistically significant, regardless of the year and group analysed.

**Table 2.** Stochastic model results.

Year/Call	2015	2017	2019	2021	All
Men	0.134***	0.154***	0.170***	0.169***	0.125***
	(0.004)	(0.004)	(0.004)	(0.003)	(0.002)
Women	0.083***	0.094***	0.111***	0.106***	0.081***
	(0.005)	(0.004)	(0.004)	(0.003)	(0.002)
Difference	0.051***	0.060***	0.058***	0.063***	0.043***
	(0.006)	(0.006)	(0.005)	(0.005)	(0.002)
Endowment	0.007***	0.010***	0.009***	0.012***	0.006***
	(0.002)	(0.002)	(0.002)	(0.002)	(0.001)
Coefficients	0.040***	0.046***	0.046***	0.048***	0.035***
	(0.006)	(0.005)	(0.005)	(0.004)	(0.002)
Interaction	0.004**	0.004**	0.003**	0.003***	0.002***
	(0.002)	(0.002)	(0.001)	(0.001)	(0.001)
Obs. Men	6479	8,132	10,374	12,773	48,398
Obs. Women	3563	4,856	6,409	8,296	28,770
Obs. Total	10,042	12,988	16,783	21,069	77,168

Source: Own elaboration.

***, ** and *indicate significance at the 1, 5 and 10%, respectively.

Specifically, the first column (year 2015) reveals that the probability of men achieving the Emeritus category is 13.4% on average, while for women it is lower and equal to 8.3%; therefore, the average gap is 5.1% as shown in the third row ( *Difference*). This differential in the probability of success is decomposed into the sum of three parts; one attributable to observable variables or more precisely those included in the stochastic model as explanatory variables ( *Endowment*) and one to unobservable variables ( *Coefficients*). The last row is a mixture of the last two ( *Interaction*).

In this order of considerations, the decomposition of the gap of 5.1% reveals that a very low part corresponds to the differences in the observable characteristics (0.7%); while a very high part corresponds to the unobservable variables (4.0%) and another very low part equal to 0.4% is attributable to the interaction. With the above, it can be affirmed that for the year 2015, there are gender gaps that are minimally explained by observable reasons and largely by gender discrimination.

The following calls (2017, 2019, 2021) repeat the previous pattern, as the possibility of reaching the highest category is always higher for men than for women, so the gap is always positive. Again, the part corresponding to the differences in the observable variables is minimal (all less than 1%); however, the difference in the coefficients that are indexed to gender discrimination phenomena is increasing, as they go from 4% in 2015 to 4.6% in 2017, remains the same for 2019; and by 2021 they reach 4.8%. This means that gender discrimination is increasing in order to reach the highest category of research in Colombia.

## Discussion

The results show that the breakdown of the gender gap between male and female researchers is 5.8%. This figure is minimally explained by observable reasons and is largely attributable to gender discrimination. This occurs in Ibero-America, where gender gaps are marked not only by the Matilda effect, which accounts for the systematic discrimination of women in science whose contributions are made invisible and assumed by their male counterparts ( Rossiter, 1993), but also by the Curie complex that exposes women scientists to an almost mythical Marie Curie benchmark of excellence, to the point of making them feel inferior and unable to reach and equal ( Des Jardins, 2010); or by the impostor syndrome ( Rose Clance & Ament Imes, 1978) where the situation has become so internalised that they believe their achievements are exaggerated and therefore normalise discrimination to the point where they feel it does not exist.

Achieving the Emeritus status requires an age of over 65 years and the chance of being Emeritus is 5.1% higher for female researchers than for their male counterparts. These differences can be explained by the time limitations that women researchers have to be productive in that they spend more time than male researchers on care work, either due to maternity or caring for other dependent family members because women are traditionally responsible for these activities, low male co-responsibility, deficiencies in the system to promote conciliation mechanisms ( Ayala del Pino, 2015) that facilitate the insertion of a new model of productivity and in Colombia is certified as family-responsible companies ( Ministerio de trabajo, 2018).

Likewise, the late or slow start of women’s research careers due to motherhood, the use of time for care work, and the fact that men retire five years later than women ( Ley 100 de 1993-Gestor normativo, 1993) contribute to the gap for women researchers to reach the Emeritus category in Colombia. The results show that for every three men in the Emeritus research category there is only one woman, and this pattern of participation is repeated in all research categories, a situation that calls for actions to support the funding of research projects and agendas led by women researchers as a gender balance strategy ( Tacsir et al., 2014).

The growth trend of researchers with doctoral education in calls for proposals is growing at decreasing rates and in regions such as Chile and Colombia the gap is 33% and 37% respectively; this situation ends up being reflected in the productivity of female researchers, where Colombia also stands out with marked gender gaps ( Albornoz et al., 2018). In this regard, the results of ( Muñoz-Ñúngo et al., 2022) indicate that in 2019, women sign 48.52% of scientific production and reinforce the trend that delays and female researchers from achieving the highest research category, Emeritus.

The results also showed that most of the researchers in Colombia are concentrated in the Social Sciences, around 30% and female participation here is the highest among the disciplinary fields 33.68% in the call for 2021, in accordance with the results of research that also show that in Colombia, the number of female researchers with similar productivity to male researchers is growing ( Ávila-Toscano et al., 2019) which reinforces the idea that women are concentrated in areas of knowledge considered not very significant ( Gaceta UNAM, 2021).

In the field of STEM, gender gaps in mathematics and engineering are around 60% and in science and technology around 25%, a situation that is worrying because the skills and knowledge of today and the future are forged in these fields, in particular, information and communication technologies (ICT) are strongly masculinised and vertical and horizontal barriers persist ( López-Bassols et al., 2018) which makes it necessary to deconstruct the stereotypes of the professions as well as to rethink the mechanisms of access, participation, promotion and advancement in these disciplines, given that they were formulated under patriarchal and masculinising perspectives ( Monroy, 2019) in consideration of the fact that these professions are better paid and the absence or low participation of women in them widens social gaps and increases the feminisation of poverty ( Goldin & Katz, 2009; Palacios Riaño, 2019).

The most relevant universities in the country reflect a greater participation of women researchers, a situation that is beginning to change with the emergence of regional universities, but, as Gutiérrez (2012) points out, the research groups led by women are classified in the lowest categories and are mainly concentrated in Bogota, Antioquia and Valle del Cauca.

## Conclusions

The results obtained allow us to affirm that there is a gender gap in scientific research in Colombia in the Emeritus research category in the calls for proposals for the period 2015-2021. Moreover, the existing gap cannot be explained by factors associated with attributes of education and academic productivity that are part of the regulatory requirements, insofar as not being explained by them, it evidences the existence of discrimination against women researchers to access the highest research category.

### Ethical aspects

The present research has received approval from the Ethics and Scientific Integrity Committee of Norbert Wiener University, under file number 298 of January 25, 2022 (298-2022). It is important to highlight that this study falls under the category of minimal risk, as it does not involve the participation of human subjects but is instead based on the collection and analysis of data from sources available online. The use of secondary information has been carried out with strict adherence to confidentiality, through the implementation of rigorous analysis codes. Due to this methodology and the nature of the data employed, it has not been necessary to obtain informed consent from individuals, as previously established. It is worth emphasizing that, despite the absence of direct interaction with participants, the study has undergone thorough ethical scrutiny by the relevant committee, which has determined that it presents minimal risk. Therefore, informed consent was not required, as mentioned earlier, given that it originates from secondary sources. However, it is important to note that the study has been approved by an ethics committee and has been deemed to pose minimal risk, as detailed in the ethical considerations.

## Data Availability

Zenodo: The Gap that Matilda will bridge: a look at the Colombian case [Data set]. Zenodo. https://doi.org/10.5281/zenodo.10806352 ( Rivera-Lozada et al., 2024) This project contains the following underlying data:
•
The Gap that Matilda will bridge.pdf Data are available under the terms of the Creative Commons Attribution 4.0 International license (CC-BY 4.0).

## Footnotes

^1^
Data retrieved from: https://minciencias.gov.co/ciudadano/datosabiertos, last accessed February 13th, 2024.

^2^
Despite this, the authors used panel data information but the results were inconclusive. These can be provided upon request to the authors.

^3^
The definition of model variables are included in Appendix 1.
