# Supplementary material for: The gap that Matilda will bridge: a look at the Colombian case
Source: F1000Res. 2024 Jun 6;13:588. [Version 1] doi: 10.12688/f1000research.147674.1 (PMC11196936; doi:10.12688/f1000research.147674.1)
Supplement: Peer review history

# Reviewer report: Reviewer response for version 1

**DOI:** 10.5256/f1000research.161898.r288271

**Published:** 2024-06-25

**Authors:** Yolvi Javier Ocaña Fernandez

**Affiliations:** 1 UNIVERSIDAD NACIONAL MAYOR DE SAN MARCOS, Lima, Peru

The manuscript titled "The Gap that Matilda Will Bridge: A Look at the Colombian Case" is a highly interesting article that seeks to identify the gaps among researchers in a country emerging in the field of research.

 The introduction is well-structured, fulfilling all essential elements, and clearly presents the situation of researchers in Colombia. The knowledge gap is precisely defined. Additionally, a detailed literature review is included, which helps the reader understand the issues faced by researchers in the Colombian context.

 Regarding methodological aspects, the appropriate Oaxaca-Blinder-Kitakagwa model has been used. This model corrects the sample selection bias by including the inverse Mills ratio (Heckman's Lambda) in an ordered probit model, ensuring the validity, reliability, and generalizability of the data. Including data from 2015 to 2021 allowed for a robust analysis.

 The results met the research objectives, particularly in identifying the gender gap between male and female researchers. It is highlighted that one needs to be over 65 years old to achieve emeritus status. Additionally, the research explains the time constraints faced by female researchers, as they dedicate more time to family responsibilities.

 The conclusions are well-formulated and align with the research objectives. It is mentioned that the existing gap cannot be explained by factors associated with education and academic productivity attributes, which are part of normative requirements. This indicates discrimination against female researchers in achieving the highest research category.

 Finally, the bibliographic references are relevant and updated concerning the topic addressed. In terms of form, the writing is clear, concise, and coherent.

 I consider this research to be a valuable contribution to gender studies, and as a reviewer, I would APPROVE it.

Is the work clearly and accurately presented and does it cite the current literature?

Yes

If applicable, is the statistical analysis and its interpretation appropriate?

Yes

Are all the source data underlying the results available to ensure full reproducibility?

Yes

Is the study design appropriate and is the work technically sound?

Yes

Are the conclusions drawn adequately supported by the results?

Yes

Are sufficient details of methods and analysis provided to allow replication by others?

Yes

Reviewer Expertise:

Education, innovation, education and research policies

I confirm that I have read this submission and believe that I have an appropriate level of expertise to confirm that it is of an acceptable scientific standard.

# Reviewer report: Reviewer response for version 1

**DOI:** 10.5256/f1000research.161898.r288264

**Published:** 2024-06-24

**Authors:** Yenny Naranjo Tuesta

**Affiliations:** 1 Tecnologico de Monterrey, Monterrey, Nuevo Leon, Mexico

The article raised the problem of women in research and especially that of achieving Emeritus status in Colombia. Although it focuses on showing the aspects related to the Matilda and/or Curie effect, the research evidence that not only that is relevant, but it also evidences that public policies also influence and do not reflect support to improve the competences and participation of women in the STEM field itself. 

 The article in general is very good and reflects this problem that not only occurs in Colombia but surely extends to other contexts. I consider that in the conclusion it falls very short and should be more emphatic in the way forward and the importance of this research for different parties.Authors are encouraged to review citations prior to indexing. 

  The article is interesting, complete and pertinent. For minor suggestions, it is recommended to review the citations before indexing, just to confirm that the cross-reference works and is correct.

Is the work clearly and accurately presented and does it cite the current literature?

Yes

If applicable, is the statistical analysis and its interpretation appropriate?

Yes

Are all the source data underlying the results available to ensure full reproducibility?

Partly

Is the study design appropriate and is the work technically sound?

Yes

Are the conclusions drawn adequately supported by the results?

Partly

Are sufficient details of methods and analysis provided to allow replication by others?

Yes

Reviewer Expertise:

My research has been focused on the field of accounting and finance. In particular sustainable accounting, accounting education, management control and sustainability.

I confirm that I have read this submission and believe that I have an appropriate level of expertise to confirm that it is of an acceptable scientific standard.

# Reviewer report: Reviewer response for version 1

**DOI:** 10.5256/f1000research.161898.r288265

**Published:** 2024-06-24

**Authors:** Ernesto Mesa-Vázquez

**Affiliations:** 1 Universidad de Almeria, Almería, Andalusia, Spain

After reading the aforementioned article, I would say that:

 I confirm that the literature review is sufficient to deal with the research problem.

 Besides, I completely agree with the authors' chosen approach. Due to the nature of the data and the research question, given the limited number of years, the Oaxaca-Blinder-Kitagawa decomposition model is the right choice. The reasons why other approaches aren't feasible are clearly explained: "Since this study uses data from 2015 onwards, it is not possible to implement panel data models as there are so few years (T=4 periods) and an increasing number of individual units (N); then (N>T): and according to Pesaran (2015), the estimators of fixed and random effects, instrumental variables and the generalised method of moments (GMM), will be biased." This is crystal clear.

 I'm quite sure the authors also conducted other estimations, such as OLS regression or multinomial regression, but these techniques wouldn't be accurate for this purpose.

 Finally, I think this paper is necessary to understand the underlying issues at Colombian universities regarding the gender gap at the top researcher level.

 Nevertheless, I have some minor comments that could be revised before indexing:

 "The overall results reveal a gap of 5.1%, whose difference in the coefficients attributable to gender discrimination phenomena is increasing from 4% in 2015 to 4.6% in 2017, remains the same for 2019 and reaches 4.8% in 2021."

 I consider this statement should be written as "... starts from 5.1% in 2015, increases to 6.0% in 2017, then decreases to 5.8% in 2019, and finally reaches 4.3% in 2021."

 In addition, I think the last column ("All") could confuse the reader. My suggestion is to delete it or provide a better explanation of its meaning.

 In equation 1, I would recommend changing the prefixes "H" and "M" throughout the article to "M" and "W" when referring to men and women.

 In Figure 3, authors should include a chart legend for better understanding. In addition, they should also include year 2021. Lastly, the authors could consider including the "Emeritus" category in the same chart as the other categories.

Is the work clearly and accurately presented and does it cite the current literature?

Yes

If applicable, is the statistical analysis and its interpretation appropriate?

Yes

Are all the source data underlying the results available to ensure full reproducibility?

Yes

Is the study design appropriate and is the work technically sound?

Yes

Are the conclusions drawn adequately supported by the results?

Yes

Are sufficient details of methods and analysis provided to allow replication by others?

Yes

Reviewer Expertise:

Intensive agriculture, 'Gender differences in overplacement, water management, ecosystem services, sustainability, farmer attitudes, experimental and behavioural economics, social preferences, herding behaviour

I confirm that I have read this submission and believe that I have an appropriate level of expertise to confirm that it is of an acceptable scientific standard.
